# *Bacillus* spp. as potential probiotics: promoting piglet growth by improving intestinal health

**DOI:** 10.3389/fvets.2024.1429233

**Published:** 2024-07-26

**Authors:** Xiaopeng Tang, Yan Zeng, Kangning Xiong, Jinfeng Zhong

**Affiliations:** ^1^State Engineering Technology Institute for Karst Desertfication Control, School of Karst Science, Guizhou Normal University, Guiyang, China; ^2^Key Laboratory for Information System of Mountainous Areas and Protection of Ecological Environment, Guizhou Normal University, Guiyang, China; ^3^Hunan Polytechnic of Environment and Biology, College of Biotechnology, Hengyang, China

**Keywords:** *Bacillus* spp., growth performance, gut microbial, intestinal health, piglets

## Abstract

The application of *Bacillus* spp. as probiotics in the swine industry, particularly for piglet production, has garnered significant attention in recent years. This review aimed to summarized the role and mechanisms of *Bacillus* spp. in promoting growth and maintaining gut health in piglets. *Bacillus* spp. can enhance intestinal barrier function by promoting the proliferation and repair of intestinal epithelial cells and increasing mucosal barrier integrity, thereby reducing the risk of pathogenic microbial invasion. Additionally, *Bacillus* spp. can activate the intestinal immune system of piglets, thereby enhancing the body’s resistance to diseases. Moreover, *Bacillus* spp. can optimize the gut microbial community structure, enhance the activity of beneficial bacteria such as *Lactobacillus*, and inhibit the growth of harmful bacteria such as *Escherichia coli*, ultimately promoting piglet growth performance and improving feed efficiency. *Bacillus* spp. has advantages as well as challenges as an animal probiotic, and safety evaluation should be conducted when using the newly isolated *Bacillus* spp. This review provides a scientific basis for the application of *Bacillus* spp. in modern piglet production, highlighting their potential in improving the efficiency of livestock production and animal welfare.

## Introduction

Weaning is one of the key events in the life cycle of pigs. Changes in diet and environment during weaning usually result in reduced feed intake, intestinal inflammation, and imbalances in intestinal microbial composition, leading to diarrhea in weaned piglets (1–3). In practical production, the addition of antibiotics (4, 5) or high doses of zinc oxide (ZnO) (2, 6–8) to the diets has been found to effectively alleviate diarrhea in piglets after weaning and promote their growth and development. However, while acknowledging the positive effects of antibiotics and high doses of ZnO on weaned piglets, it is also necessary to recognize their negative effects. Long-term or excessive of antibiotics in swine will lead to the imbalance of pig intestinal microbiota, the increase of antibiotic-resistant pathogens, and the residue of antibiotics in pork, which will directly or indirectly harm human health (9, 10). Excessive or long-term use of high doses of ZnO may lead to dull skin and coarse hair, inhibit the growth and development of pigs, reduce the bioavailability of ZnO, and may also lead to an increase in the percentage of intestinal bacteria resistant to multiple drugs, causing waste of zinc resources and environmental pollution (11–13). The prohibition or restriction of dietary antibiotics, and the reduction of ZnO in feed have formed a broad consensus in the world. Therefore, the search for green, safe and efficient non-antibiotic additives and ZnO substitutes, such as essential oils (14), organic acids (15), prebiotics (16), and probiotics (17), and so on to improve intestinal health and reduce diarrhea of weaned piglets has become a hot field of animal nutrition.

Probiotics are live bacteria that can improve the beneficial flora of the host with single or multiple bacteria (18). Previous studies have demonstrated that probiotics can improve the immune function, enhance the ability to resist bacterial infection, regulate the balance of intestinal flora, and thus improve production performance of piglets (19–22). There are many kinds of probiotics, including *Bifidobacteria*, *Lactobacillus*, yeast, *Bacillus* spp., etc. Among these probiotics, *Bacillus* spp. has gained increasing attention as an alternative to antibiotics or ZnO due to its advantages such as heat resistance during feed granulation, resistance to low pH in the stomach, and stability at ambient temperatures (23, 24).

*Bacillus* spp. are Gram-positive aerobic or facultative anaerobic bacteria capable of producing resistant endospores (25). *Bacillus* spp. can adapt to a variety of environments and are widely distributed around the world because their spores are highly resistant to ultraviolet radiation, high temperatures, strong acids, ionizing radiation, and many toxic chemicals (26, 27), making it widely used in industrial, agricultural, medical and other fields (25, 28–30). This is also the advantage of *Bacillus* spp. as a probiotic for animals. In the field of animal nutrition and feed science, compared with other types of probiotics, *Bacillus* spp. has more advantages. First of all, in the process of feed processing, *Bacillus* spp. can resist high temperature because of the presence of spores, and therefore, *Bacillus* spp. can maintain biological activity after processing, and its spore-forming ability is beneficial for long-term storage compared to that of non- pore-forming bacteria (31, 32). For example, Amerah et al. (33) showed that Bacillus spores are resistant to pelleting temperatures of up to 90 ° C, and more than 90% of spores are still alive in feed samples. Second, because of the strong stress resistance of Bacillus spores, they can tolerate cholate and stomach acid after entering the gastrointestinal tract, and successfully colonize and play their role in the intestine (23, 24). For example, Dong et al. (34) showed that *Bacillus licheniformis* (*B. licheniformis*) WBL009 had strong acid and bile salt resistance. The survival rate of *B. licheniformis* WBL009 was 35.8% after 2 h treatment in artificial gastric juice with pH value of 2.5, and 51.6% after 12 h treatment with bile salt concentration of 0.3%.

Numerous studies have shown that *Bacillus* spp. can produce extracellular enzymes such as glycoenzyme, protease and lipase, and also has antibacterial and immune regulation effects, thereby improving the nutrient digestibility, reducing the colonization of harmful bacteria such as, *Escherichia coli* and *Clostridium perfringens* of piglets, and thus improving the growth performance of piglets (35–40). There are many types of *Bacillus* spp., including *Bacillus subtilis* (*B. subtilis*), *B. licheniformis*, *Bacillus coagulans* (*B. coagulans*), *Bacillus megaliformis* (*B. megaliformis*), *Bacillus cereus* (*B. cereus*), and so on. However, not all *Bacillus* spp. are effective probiotics, some strains cannot be used because of limitations such as poor colonization effect or virulence factors, and some *Bacillus* spp. strains have been identified as opportunistic pathogens that causing food spoilage and toxin release in the hosts (41). Therefore, although most of the *Bacillus* spp. are generally recognized as safe for animal consumption, it is necessary to summarize the application effect of different *Bacillus* spp. on piglets. In this paper, the effects of *Bacillus* spp. in piglet production were summarized from the perspective of growth promotion and health benefits, so as to provide a reference for the rational utilization of *Bacillus* spp. in swine production.

## Growth promoting effect of *Bacillus* spp. on piglets

Early weaning technology is the production method commonly used in most intensive pig farms at present. The weaning process of piglets is accompanied by a series of changes in diet, society and environment, which can easily cause weaning stress (3). The transition from high digestibility liquid milk to non-digestible solid feed is a great challenge for the immature gastrointestinal tract of the piglets, which usually causes diarrhea due to indigestion (42). Due to the combination of sudden separation from the sow, social factors, and other influences, piglets experience anxiety, leading to a reduction in feed intake or even refusal to eat (3, 42, 43). It is generally believed that the production performance of the whole pig farm is determined by the production performance of the piglets, and diarrhea is the culprit leading to the low production performance of the piglets. Therefore, the control of post-weaning diarrhea of piglets is of great significance to improve the economic benefits of pig production.

Previous studies have shown that the addition of *Bacillus* spp. can improve the intestinal health of pigs by altering the intestinal flora, thereby inhibiting the growth of pathogens, enhancing immune function, improving nutrient utilization and digestibility, reducing the incidence of diarrhea, and ultimately improving the growth performance of piglets (44–48). The growth promoting effect of single *Bacillus* spp. strains on piglets were summarized in Table 1. These studies indicated that the addition of single *Bacillus* spp. strains, including *B. subtilis* (35, 45–47, 49–61), *B. coagulans* (62–66), *B. amyloliquefaciens* (44, 67–71), *B. licheniformis* (72–76), *B. halotolerans* (77), B. toyonensis (78), *B. cereuscould* (79), and so on could promote the growth performance and effectively prevent the occurrence of diarrhea of piglets.

**Table 1:** Growth promoting effect of *Bacillus* spp. on piglets.

*Bacillus* spp.	Piglet age	Optimal added amount	Experimental period	Growth performance	References
*B. subtilis*	25-days-old	100 mg/kg or 200 mg/kg	42 days	Diarrhea rate↓	Liu et al. (35)
*B. subtilis*	28-days-old	300 g/t	42 days	FBW↑, ADG↑, G/F↑	Hu and Kim (45)
*B. subtilis*	26 ± 2 days-old	4 × 10^9^ CFU/kg or 2 × 10^10^ CFU/kg	28 days	Diarrhea index↓, ADG↑, G/F↑	Hu et al. (46)
*B. subtilis*	21-days-old	2.56 × 10^9^ CFU/kg	28 days	Diarrhea score↓, ADG↑, G/F↑	He et al. (47)
*B. subtilis*	45-days-old	0.1%	28 days	FBW↑, ADG↑	Deng et al. (49)
*B. subtilis*	25-days-old	1.44× 10^9^ CFU/kg	28 days	ADG↑, diarrhea rate↓	Tian et al. (50)
*B. subtilis*	25-days-old	500 mg/kg	42 days	ADG↑, F/G↓	Tang et al. (51)
*B. subtilis*	28-days-old	1 × 10^9^ CFU/kg	42 days	FBW↑, ADG↑, F/G↓	Wang et al. (52)
*B. subtilis*	26.9 ± 2.0 days-old	1.875 × 10^5^ CFU/g	14 days	FBW↑, ADG↑	Duddeck et al. (53)
*B. subtilis*	28-days-old	1.0× 10^9^ CFU/kg	Not mentioned	FBW↑, nitrogen deposition rate↑	He et al. (54)
*B. subtilis*	21-days-old	1 × 10^8^ CFU/mL	42 days	ADG↑, ADFI↑	Jia et al. (55)
*B. subtilis*	21-days-old	500 mg/kg	28 days	FBW↑, ADG↑, diarrhea rate↓	Júnior et al. (56)
*B. subtilis*	25 ± 2 days-old	1.0× 10^7^ CFU/kg	14 days	ADG↑, F/G↓	Li et al. (57)
*B. subtilis*	21-days-old	0.05%	21 days	G/F↑, diarrhea rate↓	Park et al. (58)
*B. subtilis*	Not mentioned	1.28 × 10^9^ CFU/kg or 2.56 × 10^9^ CFU/kg	19 days	ADG↑	Kim et al. (59)
*B. subtilis*	21-days-old	1.0× 10^9^ CFU/kg	28 days	FBW↑, ADG↑, diarrhea index↓	He et al. (60)
*B. subtilis*	Not mentioned	2.0× 10^9^ CFU/kg	28 days	ADG↑, FCR↓, diarrhea index↓	Sudan et al. (61)
*B. coagulans*	26-days-old	600 g/t	28 days	ADG↑, F/G↓, diarrhea index↓	Sun et al. (62)
*B. coagulans*	7-days-old	1.0× 10^8^ CFU/kg	7 days	ADFI↑	Zhang et al. (63)
*B. coagulans*	24-days-old	400 mg/kg	28 days	ADG↑	Fu et al. (64)
*B. coagulans*	21-days-old	2 × 10^6^ CFU/kg or 1 × 10^7^ CFU/kg	21 days	Diarrhea rate↓	Wu et al. (65)
*B. coagulans*	21-days-old	2 × 10^6^ CFU/kg	14 days	Diarrhea rate↓	Wu et al. (66)
*B. amyloliquefaciens*	28-days-old	5.0× 10^9^ CFU/kg	14 days	FBW↑, ADG↑, F/G↓, diarrhea rate↓	Du et al. (67)
*B. amyloliquefaciens*	21-days-old	2.0 g/kg	28 days	ADG↑, G/F↑, feed efficiency↑	Li et al. (68)
*B. amyloliquefaciens*	28-days-old	1.0× 10^9^ CFU/kg	32 days	ADG↑, diarrhea rate↓	Ji et al. (69)
*B. amyloliquefaciens*	42-days-old	2.0× 10^8^ CFU/kg	28 days	ADG↑, ADFI↑	Wang et al. (70)
*B. amyloliquefaciens*	21-days-old	1.0× 10^8^ CFU/kg	42 days	ADG↑, FBW↑, F/G↓, diarrhea rate↓	Wang et al. (71)
*B. licheniformis*	28-days-old	500 mg/kg	14 days	FBW↑, ADG↑, F/G↓, diarrhea rate↓	Yu et al. (72)
*B. licheniformis*	21 ± 1-days-old	1 × 10^9^ CFU/kg or 1 × 10^10^ CFU/kg	28 days	ADG↑, F/G↓, diarrhea rate↓	Cao et al. (73)
*B. licheniformis*	28-days-old	500 mg/kg or 1,000 mg/kg	28 days	ADG↑, diarrhea rate↓	Yu et al. (74)
*B. licheniformis*	21 ± 1-days-old	1,000 g/t	14 days	FBW↑, ADG↑, diarrhea incidence↓	Sun et al. (75)
*B. licheniformis*	21-days-old	2 × 10^9^ CFU/kg	17 days	Diarrhea index score↓	Xu et al. (76)
*B. halotolerans*	25-days-old	1 × 10^9^ CFU/kg	42 days	Diarrhea rate↓	Li et al. (77)
*B. toyonensis*	24 ± 3-days-old	500 mg/kg or 1,000 mg/kg	44 days	FBW↑, ADG↑, diarrhea rate↓	Kantas et al. (78)
*B. cereus*	28 ± 3-days-old	1 × 10^9^ CFU/kg	35 days	FBW↑, ADG↑, FCR↓, diarrhea score↓	Papatsiros et al. (79)

ADFI, average daily feed intake; ADG, average daily gain; FBW, final body weight; FCR, feed conversion ratio; F/G, feed in-take to gain ratio; G/F, gain to feed intake ratio. “↑” stands for increase, and “↓” stands for decrease.

In addition to the addition of single strains of *Bacillus* spp., the combination of *Bacillus* spp. with other probiotics or functional substances has shown a synergistic effect in promoting the growth performance of piglets. For example, Liu et al. (35) showed that the combination of *B. subtilis* QST713 (100 mg/kg) with β-mannanase (150 mg/kg) effectively decreased the feed conversion ratio (FCR) of piglets throughout the trial period. Jiao et al. (37) demonstrated that compared to the control group, the combination of *Bacillus* spp. (1.3 × 10^9^ CFU/kg; *B. licheniformis* and *B. subtilis* with the ratio of 1: 1) with medium-chain fatty acid (0.588 g/kg) significantly increased the average daily gain (ADG) and dry matter intake of piglets in phase 1 (days 1 to 9), and significantly increased the ADG of piglets in the whole period (days 1 to 36). Liu et al. (80) showed that the combination of *B. subtilis* with *Lactobacillus plantarum* (*L. plantarum*) jointly increased average daily feed intake (ADFI) and ADG of weaned piglets in d 14 ~ 28 and d 28 ~ 42. Pu et al. (81) showed that piglets fed a diet added with benzoic acid (3,000 g/t) and *B. coagulans* (400 g/t) mixture significantly increased the final body weight (FBW) and ADG, and decreased FCR compared with piglets fed a basal diet. Phaengphairee et al. (82) showed that dietary supplementation of full-fat black soldier fly larvae in combination with multi-probiotics (*B. subtilis*, *B. licheniformis*, and *Saccharomyces cerevisiae*) significantly increased the ADG, gain-to-feed ratio (G/F), and nutrient digestibility of weaned piglets.

Although numerous studies have confirmed the growth-promoting effect of *Bacillus* spp. alone or in combination with other additives on weaned piglets, some studies have shown that *Bacillus* spp. has no effect on the growth performance of weaned piglets. For example, Jiao et al. (37), Huting et al. (38), Kritas and Morrison (83), Luise et al. (84), and Ding et al. (85) showed that dietary supplementation with *Bacillus* spp. alone has no effect on the growth performance of piglets. The different effects of *Bacillus* spp. on alleviating diarrhea and improving growth performance of weaned piglets may be closely related to the different types of *Bacillus* spp., dosage, adding stage, use mode, diet composition and physiological health status of piglets. Although the effect of *Bacillus* spp. on the growth performance of weaned piglets is inconsistent, it is certain that *Bacillus* spp. has some other benefits, such as promoting intestinal health.

## Intestinal health benefits of *Bacillus* spp. on piglets

Weaning is the transitional period when piglets shift from relying on maternal milk nutrition to solid feed. During this time, the digestive system of the piglets faces significant challenges, making them susceptible to intestinal dysfunction and diseases (86, 87). Promoting intestinal development and intestinal health of weaned piglets is the key to cope with weaning stress. In previous studies, there are many ways to promote the intestinal health of piglets, among which probiotics, especially *Bacillus* spp., are prominent in promoting the intestinal health of piglets.

### *Bacillus* spp. promotes intestinal development

The gut is the main site for the digestion and absorption of nutrients in animals and serves as a selective barrier for the body to prevent exogenous harmful substances from entering the circulatory system (3, 87). The gastrointestinal digestive system of piglets is still immature, and external stress will further damage the intestinal structure of piglets, such as intestinal villi shedding, crypt hyperplasia, intestinal mucosal atrophy, etc., and then destroy the intestinal mucosal barrier function and digestive absorption capacity (3, 88, 89). Previous studies have shown that *Bacillus* spp. has good nutritional physiological effects on intestinal development of piglets, including the maintenance of intestinal morphology and structure (50, 71, 73, 75, 85, 90), promoting the secretion of intestinal digestive enzymes (49, 75, 91, 92), and reducing intestinal permeability (59, 90, 91).

*Bacillus* spp. have a positive effect on the intestinal morphological structure and function of piglets, and can improve the intestinal damage caused by various stresses, so as to maintain the integrity of intestinal mucosa (65, 66). *Bacillus* spp. can increase the intestinal villus height (VH), decrease the intestinal crypt depth (CD), increase the intestinal villus height to crypt depth ratio (VCR) and reduce the apoptosis of intestinal mucosal epithelial cells of piglets (50, 67, 68, 92). For instance, Du et al. (67) showed that dietary supplementation of *B. amyloliquefaciens* (5.0× 10^9^ CFU/kg) significantly increased intestinal VH and VCR, while decreased intestinal CD. Hu et al. (92) reported that dietary supplementation with *B. subtilis* PB6 can improve intestinal damage of suckling piglets caused by intrauterine growth retardation (IUGR), as shown by higher VH and VCR, and lower CD than that of IUGR piglets without *B. subtilis* supplementation. Du et al. (93) showed that dietary supplementation with *B. amyloliquefaciens* SC06 (2.0 × 10^8^ CFU/kg) significantly increased intestinal villus length, and intestinal villi morphology was improved. Wang et al. (36) showed that the combination of *B. subtilis* with *B. licheniformis* significantly increased the ileum VH and the jejunum and ileum VCR, and decreased the jejunum CD of piglets. The increase in VH and the decrease in CD on the intestinal mucosa effectively increase the surface area of the intestinal tract in contact with the digested food, thus improving the absorption efficiency of nutrients.

Most *Bacillus* spp. have a strong enzyme production capacity and can produce a variety of extracellular enzymes, such as cellulase, xylanase, amylase, protease, lipase (94–98), which can assist animals to digest feed and improve nutrient absorption. In addition to the exogenous enzymes secreted by themselves, *Bacillus* spp. can also stimulate the secretion of various digestive enzymes in the intestines of piglets (49, 75, 91, 92). For example, Deng et al. (49) showed that *B. subtilis* supplementation significantly increased ileum lipase, amylase, lactase, and maltase activities compared to control group, and significantly increased ileum lactase, and maltase activities compared to antibiotic group. Hu et al. (91) showed that replacement of aureomycin with *B. amyloliquefaciens* (2 × 10^8^ cfu/Kg) significantly improved intestinal amylase, disaccharides and Na^+^/K^+^-ATPase activities of piglets, and half replacement of aureomycin with *B. amyloliquefaciens* (1 × 10^8^ cfu/Kg), the activity of intestinal chymotrypsin was significantly improved. Many studies have shown that dietary addition of *Bacillus* spp. can significantly improve the nutrient digestibility of piglets (38, 45, 99–101), which may be related to the secretion of intestinal digestive enzymes. For instance, Huting et al. (38) showed that dietary addition of multi-strains of *Bacillus* spp. significantly improved the apparent total tract digestibility (ATTD) of dry matter (DM) and organic matter (OM) of piglets. Hu and Kim (45) showed that dietary *B. subtilis* C-3102 supplementation significantly improved the apparent the ATTD of DM, crude protein (CP), and energy of piglets. Cai et al. (99) and Lewton et al. (100) showed that piglets fed with *Bacillus* spp. based direct-fed microbial had a higher protein utilization, as indicated by increased the ATTD of nitrogen. Cui et al. (101) showed that the ether extract (EE) and phosphate digestibility was increased when piglets fed with *B. subtilis*.

When exposed to stress conditions such as weaning stress, *Escherichia coli* (*E. coli*) infection, IUGR and lipopolysaccharide (LPS) stimulation, piglets are susceptible to intestinal mucosal injury, potentially leading to increased intestinal permeability (3, 87, 102–104). In general, intestinal permeability is assessed by quantifying the passage of small molecules like horseradish peroxidase (59), monitoring plasma levels of D-lactic acid, endotoxins, and diamine oxidase (DAO) (37, 44, 105), as well as by measuring trans-epithelial electrical resistance (TEER) in intestinal tissues (106). Studies have shown that *Bacillus* spp. can reduce intestinal permeability of piglets. Kim et al. (59) demonstrated that a high dosage of *B. subtilis* (2.56 × 10^9^ CFU/kg) supplementation lowered both intestinal intercellular and intracellular permeability in *E. coli* infected pigs. Hu et al. (91) showed that substituting aureomycin with *B. amyloliquefaciens* (2 × 10^8^ cfu/Kg) preserved intestinal integrity, as evidenced by significantly decreased DAO activity. Xie et al. (107) showed that a combination of *Lactobacillus acidophilus* and *B. subtilis* reduced serum DAO level in piglets. In an IPEC-J2 cell model of deoxynivalenol (DON)-induced injury, Gu et al. (108) reported that TEER was higher in cells treated with *B. subtilis* compared to non-*B. subtilis* treated cells.

### *Bacillus* spp. promotes intestinal mucosal barrier

The intestinal epithelium serves as the primary barrier, facilitating nutrient breakdown and absorption via brush border enzymes and various transport proteins at the apical and basolateral membranes, while also protecting against antigenic invasion (3). Tight junctions are vital components of the intestinal mucosal barrier against harmful pathogens, predominantly made up of membrane protein complexes, including zonula occludens proteins (ZOs), occludin, and claudins (109). Alterations in the structure and function of tight junctions may directly impair the integrity of the intestinal mucosal barrier, leading to the infiltration of pathogens and other undesirable substances through the epithelial layer (110). Previous studies have confirmed the regulatory role of *Bacillus* spp. on intestinal tight junction proteins in piglets (35, 75, 105, 111–113). For instance, in a study using an IPEC-J2 cells model infected with *E. coli*, Sudan et al. (37) demonstrated that *B. subtilis* significantly upregulated the expression of *ZO-1*, *claudin-1*, and *occludin* genes. Similarly, Li et al. (77) found that *B. halotolerans* markedly increased both the gene and protein expression levels of ZO-1, claudin-1, and Occludin in weaned piglets suffering from *E. coli*-induced diarrhea. Sun et al. (75) showed that dietary inclusion of *B. licheniformis* significantly promoted the expression of Occludin and ZO-1 in jejunum mucosa of piglets. Additionally, Fu et al. (105) demonstrated that piglets supplemented with *B. licheniformis*, either alone or in combination with *Clostridium butyricum* significantly up-regulated the protein expression of ZO-1 and Occludin in the jejunum, as well as ZO-1, Claudin-1 and Occludin in the ileum of piglets. In addition, Yang et al. (114) showed that dietary supplementation with a low dose (3.9 × 10^8^ CFU/day) of a mixture of *B. licheniformis and B. subtilis* significantly up-regulated the protein expression of ZO-1 in the jejunum of piglets infected with *E. coli*, thereby preventing the loss of intestinal epithelial barrier integrity.

Another important molecule in the intestinal mucosal barrier is mucins (MUCs), which are secreted by intestinal goblet cells (87, 115). As principal constituents of the mucus layer, MUCs facilitate gut lubrication and form the initial line of defense within the mucosal barrier, which can promote the colonization of symbiotic bacteria, inhibit the attachment of pathogens, and maintain the homeostasis of the intestinal environment (116, 117). Therefore, the differentiation and proliferation of intestinal goblet cells, along with the normal secretion of MUCs, are crucial for maintaining intestinal health. Stress commonly disrupts these processes in piglets, leading to compromised cellular functions. For example, studies by Li et al. (77), Zhang et al. (118), and Xu et al. (119) showed that the number of intestinal goblet cells decreased significantly when piglets were infected with *E. coli*. Conversely, *Bacillus* spp. have been shown to enhance the maturation and differentiation of intestinal goblet cells and the secretion of MUCs in piglets (60, 68, 80, 93, 107, 118). Specifically, research by He et al. (60) revealed that dietary inclusion of *B. pumilus* significantly increased the number of goblet cells in duodenal villi, while supplementation of *B. subtilis* significantly up-regulated the expression of *MUC2* gene in the jejunum mucosa of piglets. Liu et al. (80) showed that the combination of *B. subtilis* with *L. plantarum* at dosage of 1 kg/t significantly increased the number of colonic goblet cells of weaned piglets. Zhang et al. (118) demonstrated that dietary supplementation a dosage of 7.8 × 10^8^ CFU/kg *Bacillus* spp. probiotics mixture significantly increased the number of ileal goblet cells, and up-regulated the expression of *MUC2* gene in the ileum of piglets infected with *E. coli*. In conclusion, the protective effect of *Bacillus* spp. on intestinal mucosal barrier of piglets is achieved by promoting the expression of intestinal tight junction proteins and the secretion of MUCs.

### *Bacillus* spp. promotes intestinal immune function and inhibits inflammatory response

The intestine is not only the most important digestive organ in animals but also the largest immune organ. The gut immune system recognizes and combats pathogens, such as bacteria, viruses, and parasites, that traverse the gut surface to prevent them from entering the bloodstream and causing systemic infections, thus playing a pivotal role in maintaining overall health (3). The intestinal development of piglets is still immature, and piglets will encounter many pathogenic and non-pathogenic challenges due to physiological and psychological factors during weaning, leading to the destruction of intestinal immune barrier function (3, 120). Numerous *in vitro* and *in vivo* studies have confirmed that *Bacillus* spp. can enhance the intestinal immune function of piglets by promoting the proliferation of immune cells, enhancing immune function, and inhibiting inflammatory responses (44, 47, 59, 114, 121).

Initially, *Bacillus* spp. enhances the intestinal immune capacity of piglets by stimulating the activity and proliferation of immune cells. The intestinal immune system of piglets is not fully matured in their early stages, and *Bacillus* spp. can stimulate immune cells in the intestinal submucosa, particularly T cells and B cells in the gastrointestinal-associated lymphoid tissue (GALT), to promote their proliferation and differentiation (114, 122, 123). This proliferation helps to establish a stronger and more active immune defense network, thereby enhancing the ability to resist pathogenic invasions. For instance, Xie et al. (107) showed that dietary supplementation with *L. acidophilus* and *B. subtilis* mixture increased CD4+ T cells and sIgA+ cells in intestinal mucosa of piglets, and Zhang et al. (121) showed that oral administration of *B. subtilis* significantly increased the number of IgA secreting cells and CD3+ T cells in intestinal tract of piglets, which suggests a direct influence on the mucosal immune response. Yang et al. (114) demonstrated that dietary supplementation with high-dose (7.8 × 10^8^ CFU/day) of *B. licheniformis* and *B. subtilis* mixture significantly increased the percentage of CD4^−^CD8^−^T cells in the inflamed intestine of piglets challenged with *E. coli*. Similarly, Zhou et al. (122) showed that adding a combination of *B. licheniformis* and *B. subtilis* to the diet notably increased the percentage of CD4 + Foxp3+ T regulatory cells among the intraepithelial lymphocytes. It also increased the presence of CD4 + IL-10+ T cells in the Peyer’s patches and the lamina propria of small intestines in *E. coli*-infected piglets.

Secondly, *Bacillus* spp. enhances the intestinal immune function of piglets by producing a variety of antimicrobial substances, such as antimicrobial peptides (AMPs) and short-chain fatty acids (SCFAs), which directly inhibit the growth of pathogens, thereby protecting piglets from infections (77, 105, 107, 124). For example, Xie et al. (107) observed that dietary inclusion of a combination of *L. acidophilus* and *B. subtilis* upregulated the gene expression of AMPs, including porcine beta defensin-2 (*PBD-2*), *PBD-3* and regenerating islet-derived IIIγ (*RegIIIγ*), and short-chain fatty acid receptors including *GPR43*, *GPR41*and *GPR109A* in the ileum mucosa of piglets. Fu et al. (105) showed that the use of *B. licheniformis* alone had no effect on the expression of AMPs genes, while the combination of *B. licheniformis* and *Clostridium butyricum* could significantly increase the expression of intestinal AMPs genes, including *PBD-1*, *PBD-2*, *PBD-3* and *PR-39* in piglets. At the same time, the use of *B. licheniformis* alone or in combination with *Clostridium butyricum* significantly increased the content of acetic, propionic acid, butyric acid, and total acid in the ileal contents of piglets.

Finally, *Bacillus* spp. also plays an important role in inhibiting inflammation by regulating the production of inflammatory cytokines, such as reducing the release of pro-inflammatory factors (such as TNF-α, IL-1β, and IL-6), while increasing the production of anti-inflammatory cytokines (such as IL-10, and IL-22), thereby preventing intestinal damage caused by excessive immune activity (47, 59, 72, 80, 105, 107). For example, Yu et al. (72) showed that dietary *B. licheniformis* supplementation significantly increased anti-inflammatory factors (IL-10), and reduced pro-inflammatory factors (TNF-α and IL-6) levels in the jejunal mucosa of piglets challenged with LPS. Li et al. (77) showed that *B. halotolerans* can inhibit the expression of various inflammatory factors in intestines of piglets by suppressed the activation of the toll-like receptor (TLR)2/TLR4-myeloid differentiation factor 88 (MyD88)-nuclear transcription factor-κB (NF-κB) pathway. Xie et al. (107) demonstrated that dietary inclusion of a combination of *L. acidophilus* and *B. subtilis* upregulated the expression of IL-22 in the ileum mucosa of piglets. In an *E. coli*-infected IPEC-1 model, Ji et al. (69) showed that *B. amyloliquefaciens* can inhibit the mRNA expression of *IL-1α*, *IL-6*, *IL-8*, and *TNF-α* by suppression of mitogen-activated protein kinase (MAPK) signaling pathways. In addition, *Bacillus* spp. can promote intestinal immune function by promoting the secretion of intestinal secretory immunoglobulin A (sIgA), which is also an important mechanism for suppressing excessive immune responses and inflammation (44, 77, 80, 107).

### *Bacillus* spp. inhibits pathogenic bacteria and regulates intestinal flora homeostasis

The gastrointestinal tract of animals is inhabited by a large number of microorganisms, and these microorganisms and their metabolites play an important role in host health in terms of nutrition, intestinal barrier and immunity through interaction with intestinal mucosa (125, 126). Changes in diet, environment, and other factors impact (3, 87). The optimization of intestinal microbial structure of piglets by nutritional strategies is one of the current research hotspots (127–129). Among which, the use of probiotics including *Bacillus* spp. have a significant effect on the regulation of intestinal microbial homeostasis in piglets (76, 130–133). For example, Wang et al. (36) showed that dietary supplementation with *B. subtilis* and *B. licheniformis* mixture decreased the abundance of *Blautia* and *Clostridium*, while increased the abundances of *Bacteroidetes* and *Ruminococcaceae*. Hu et al. (46), Wang et al. (52), and Li et al. (77) showed that piglets fed *Bacillus* spp. significantly increased the number of intestinal *Lactobacillus* and decreased the number of *E. coli*. In which, *Bacteroidetes* is benefits for the degradation of proteins and carbohydrates (134), and the activation of host’s immune system (135). *Ruminococcaceae* are associated with energy production and can ferment cellulose and hemicellulose to produce SCFAs (136). *Clostridium* is associated with diarrhea, and high abundance of intestinal *Clostridium* increases the risk of diarrhea in piglets (137). The abnormal increase of *E. coli* abundance can cause intestinal oxidative damage, reduce immune function and destroy intestinal integrity, making it a primary pathogen causing diarrhea of piglets (77, 138). *Lactobacillus* plays an important role in intestinal health, such as preventing diarrhea and intestinal infections, so it is considered to be a beneficial bacterium to maintain the balance of intestinal flora (46). Previous studies shown that an important sign of piglet diarrhea is primarily distinguished by the increased number of *E. coli* and the decreased number of *Lactobacillus* in intestines (46, 77, 138).

Therefore, the potential mechanisms by which *Bacillus* spp. modulate the gut microbial composition of piglets involve the promotion of beneficial bacterial proliferation and the suppression of pathogenic bacterial growth. On one hand, *Bacillus* spp. competes with opportunistic pathogens for adhesion sites and nutrients, thereby inhibiting the attachment and colonization of harmful microbes in the intestinal tract (139). On the other hand, *Bacillus* spp. ferments carbohydrates to produce a large amount of L-lactic acid, which reduces intestinal pH value, forms an anaerobic acidic environment conducive to the growth of beneficial bacteria such as *Lactobacillus* and *bifidobacterium*, and prevents the invasion of aerobic and eosinophilic pathogens (140, 141). In addition, *Bacillus* spp. secretes a variety of antibacterial substances, such as MUCs, AMPs, sIgA, and SCFAs, which exhibit marked antagonistic activity against various pathogens, thereby regulating the balance of intestinal microbiota (80, 107, 118).

The beneficial effects of *Bacillus* spp. on the intestinal health of piglets are summarized in Table 2. A review of these literatures revealed that *Bacillus* spp. exert a positive influence on intestinal health of piglets. Firstly, *Bacillus* spp. facilitates intestinal development and reduces gut permeability in piglets. Secondly, *Bacillus* spp. enhances the function of the intestinal mucosal barrier by upregulating tight junction proteins and stimulating MUCs secretion. Thirdly, *Bacillus* spp. enhances intestinal immunity by activating immune cells within the gut, modulating the secretion of both pro-inflammatory and anti-inflammatory cytokines, and increasing the production of secretory immunoglobulins. Lastly, *Bacillus* spp. helps maintain a dynamic balance of the intestinal microbiota by encouraging the growth of beneficial microorganisms and suppressing the proliferation of pathogenic bacteria.

**Table 2:** Beneficial effects of *Bacillus* spp. on the intestinal health of piglets.

*Bacillus* spp.	Optimal added amount	Intestinal health benefits	References
*B. subtilis*	100 mg/kg or 200 mg/kg	E-cadherin, ZO-1, and occludin↑, *E. coli* and *Clostridium perfringens*↓	Liu et al. (35)
*B. subtilis* and *B. licheniformis* mixture	4 × 10^9^ CFU/g	VH and VCR↑, CD↓, TLR-4 and TNF-α↓, *Bacteroidetes* and *Ruminococcaceae*↑, *Blautia* and *Clostridium*↓	Wang et al. (36)
*B. subtilis* and *B. licheniformis* mixture	1.3 × 10^9^ CFU/kg	Intestinal permeability↓, Shannon and Simpson index↑, *Treponema*↑	Jiao et al. (37)
*B. amyloliquefaciens*	1.0× 10^9^ CFU/kg	Intestinal permeability↓, IL-1β and IFN-γ↓, T-AOC, IL-10 and sIgA↑, *Bacteroides*, *Phascolarctobacterium* and *Desulfovibrio*↑, *Streptococcus, Tyzzerella, Vellionella* and *paraeggerthella*↓	Jiang et al. (44)
*B. subtilis*	4 × 10^9^ or 2 × 10^10^ CFU/kg	*E. coli*↓, *Lactobacillus*↑	Hu et al. (46)
*B. subtilis*	2.56 × 10^9^ CFU/kg	Claudin 1↓, IL-6 and PTGS2↓	He et al. (47)
*B. amyloliquefaciens*	2.56 × 10^9^ CFU/kg	Firmicutes and *Bifidobacterium*↑, Actinomycetota, Bacteroidota and *Clostridium sensustricto* 1↓	Jinno et al. (48)
*B. subtilis*	0.1%	Lipase, amylase, and maltase activities↑, VCR↑, Firmicutes↑, *E. coli*↓	Deng et al. (49)
*B. subtilis*	1.44× 10^9^ CFU/kg	VH and VCR↑, *Anaerovibrio* and *Bulleidia*↑, *Clostridium* and *Coprococcus*↓, colonic SCFAs↑	Tian et al. (50)
*B. subtilis*	500 mg/kg	VH and VCR↑, propionic acid and butyric acid↑, *occludin*, *EGF*, and *IGF-1R*↑, *Bacillus* and *Bifidobacterium*↑	Tang et al. (51)
*B. subtilis*	1 × 10^9^ CFU/kg	*Lactobacillus*↑, *E. coli*↓	Wang et al. (52)
*B. subtilis*	1.875 × 10^5^ CFU/g	*E. coli*↓, butyrate↑	Duddeck et al. (53)
*B. subtilis*	1.0× 10^9^ CFU/kg	Pedicoccus, Collinella, Turiciator, Veillonella, Clostridium, and Escherichia in jejunum↑, Olsenella and Pediococcus in ileum↑	He et al. (54)
*B. subtilis*	1 × 10^8^ CFU/mL	Intestinal permeability↓, GPx and SOD↑, MDA and H_2_O_2_↓, *Occludin*↑	Jia et al. (55)
*B. subtilis*	500 mg/kg	VH and VCR↑, intestinal permeability↓	Júnior et al. (56)
*B. subtilis*	1.0× 10^7^ CFU/kg	Serum IFN-γ and TNF-α↓, serum IL-10, IgA and IgG↑	Li et al. (57)
*B. subtilis*	0.05%	VH and VCR↑	Park et al. (58)
*B. subtilis*	1.28 × 10^9^ CFU/kg or 2.56 × 10^9^ CFU/kg	Intestinal permeability↓, *MUC2*↑, IL-1α and IL-6↓	Kim et al. (59)
*B. subtilis*	1.0× 10^9^ CFU/kg	Total coliforms in mesenteric lymph nodes↓, *MUC2*↑, *PTGS-2* and *IL-1β*↓, *Lachnospiraceae, Peptostreptococcaceae* and *Pasteurellaceae*↓	He et al. (60)
*B. subtilis*	2.0× 10^9^ CFU/kg	lactic acid bacteria and *Bacillus* spp.↑, *E. coli* and total *coliforms*↓, IL-8↓, MUC-1 and occludin↑	Sudan et al. (61)
*B. coagulans*	600 g/t	Bacteria diversity↑, *Ruminococcaceae_UCG-014*↑, *Prevotella_9*, *unclassified_f__Lachnospiraceae*, and *Anaerovibrio*↓	Sun et al. (62)
*B. coagulans*	1.0× 10^8^ CFU/kg	VH and VCR↑, MPO and apoptosis of intestinal epithelial cells↓, *Enterococcus*, *Clostridium* and *Lactobacillus* in jejunum↓, *E. coli*, *Bifidobacterium* and *Lactobacillus* in colon↓	Zhang et al. (63)
*B. coagulans*	400 mg/kg	Serun C3, LZM and TNF-α↓, VCR↑, *ZO-1*↑, IL-1β↓, *TNF-α*↓	Fu et al. (64)
*B. coagulans*	2 × 10^6^ CFU/kg or 1 × 10^7^ CFU/kg	Intestinal permeability↓, VH ↑, CD↓, SOD and CAT↑, MDA and H_2_O_2_↓	Wu et al. (65)
*B. coagulans*	2 × 10^6^ CFU/kg	Intestinal morphology↑, Occludin↑, GSH-Px↑, MDA↓, serum TNF-α, IL-1β↓, caspase-3↓	Wu et al. (66)
*B. amyloliquefaciens*	5.0× 10^9^ CFU/kg	*Lachnospiraceae*, *Peptococcaceae.rc4_4*, *Erysipelotrichaceae.L7A_E11*, and *Mollicutes.RF39*↑	Du et al. (67)
*B. amyloliquefaciens*	2.0 g/kg	VH and VCR↑, goblet cell↑, IL-10↑, TNF-α↓, *Lactobacillus* and *Bifidobacterium*↑, *E. coli*↓	Li et al. (68)
*B. amyloliquefaciens*	2.0× 10^8^ CFU/kg	MDA↓, T-AOC and GSH-Px↑, *SOD*, *CAT*, *GST* and *NQO1*↑, Nrf2↑	Wang et al. (70)
*B. amyloliquefaciens*	1.0× 10^8^ CFU/kg	Goblet cells↑, VH and VCR↑, IL-10↑, TNF-α↓, *Lactobacillus*↑	Wang et al. (71)
*B. licheniformis*	500 mg/kg	Serum IL-10↑, serum IL-1β and IL-6↓, acetic acid, propionic acid, butyric acid, isobutyric acid, and isovaleric acid↑, *Clostridium_sensu_stricto_1* and *Oscillospira*↑	Yu et al. (72)
*B. licheniformis*	1 × 10^9^ CFU/kg or 1 × 10^10^ CFU/kg	Serum IgA, IgG, and IgM↑, VCR↑, propionic and isobutyric acid↑, *Prevotella*↑	Cao et al. (73)
*B. licheniformis*	500 mg/kg or 1,000 mg/kg	IgA and IgG↑, IL-1β↓, acetate and propionic acids↑	Yu et al. (74)
*B. licheniformis*	1,000 g/t	VH and VCR↑, *SOD1*, *Nrf2*, and *HO-1*↑, sIgA, occludin and ZO-1↑, *Lactobacillus*↑, *clostridium_sensu_stricto_1*↓	Sun et al. (75)
*B. licheniformis*	2 × 10^9^ CFU/kg	*Aminopeptidase N*, *occludin*, *ZO-1*, and *SGLT1*↑, *Bacteroidetes*↓	Xu et al. (76)
*B. halotolerans*	1 × 10^9^ CFU/kg	VH and VCR↑, intestinal permeability↓, ZO-1, claudin-1 and occludin↑, sIgA↑, *Lactobacillus*↑, *E. coli*↓	Li et al. (77)
*B. toyonensis*	500 mg/kg or 1,000 mg/kg	Lactic acid bacteria↑, enteric pathogens↓	Kantas et al. (78)
*B. subtilis* and *L. plantarum* mixture	1 kg/t, 1.5 kg/t, or 2 kg/t	VCR↑, goblet cells↑, intestinal permeability↓, IL-1β, TNF-α, and IL-2↓, sIgA↑, SOD↑, MDA↓, *Claudin-1*, *occludin*, *ZO-1* and *MUC1*↑, *Lactobacillus*↑	Liu et al. (80)
*B. coagulans* and benzoic acid mixture	400 g/t and 3,000 g/t, respectively	Serum TNF-α↓, *SGLT1*, *claudin-1*, *occludin* and *mucin2*↑, *Bifidobacterium* and *Bacillus*↑	Pu et al. (81)
*B. subtilis*	1.28 × 10^6^ CFU/g	Villus mitotic index↑	Luise et al. (84)
*B. subtilis*	500 g/t	VH and VCR↑, butyrate, tryptamine, and cadaverine↑, skatole↓, Phylum Cyanobacteria and Proteobacteria↑, Genus *Actinobacter*, *Coprococcus*, *Enterococcus*, and *Dorea*↑	Ding et al. (85)
*B. coagulans* and *lactulose* mixture	2 × 10^9^ CFU/kg and10 g/kg, respectively	VH and VCR↑, apoptosis↓, ZO-2, Occludin and claudin-3 protein↑	Zheng et al. (90)
*B. amyloliquefaciens*	2 × 10^8^ CFU/kg	Intestinal permeability↑, amylase, disaccharides and Na^+^/K^+^-ATPase↑, β-diversity of intestinal microbiota↑	Hu et al. (91)
*B. amyloliquefaciens*	2 × 10^8^ CFU/kg	Villus length↑, VCR↑, goblet-cell number↑, tight junction proteins↑, *TNF-α* and IL-1α↓	Du et al. (93)
*B. subtilis*	2 × 10^9^ CFU/kg	VCR↑, intestinal permeability↓, *ZO-1*, *occludin*, and *claudin-1*↑, protein carbonyl↓, CAT and SOD↑, *SOD1*, *CAT* and *Nrf2*↑, HO-1, SOD1, and Nrf2↑	Yun et al. (104)
*B. licheniformis*	1 × 10^9^ CFU/kg	Intestinal permeability↓, ZO-1, claudin −1 and occludin↑, *Phascolarctobacterium*↓	Fu et al. (105)
*B. subtilis* and *L. acidophilus* mixture	0.1%	Intestinal permeability↑, ZO-1, Occludin, and MUC2↑, *PBD-2*, *PBD-3*, and *RegIIIγ*↑, CD4+ T cells and sIgA+ cells↑, sIgA↑, IL-22↑	Xie et al. (107)
*L. johnsonii, B. licheniformis* and *B. subtilis* mixture	1 × 10^8^, 4 × 10, and 4 × 10^5^ CFU/mL, respectively	Goblet cells↑, cell death↓, *Salmonella*↓	Liu et al. (112)
*B. licheniformis* and *B. subtilis* mixture	3.9 × 10^8^ CFU/day or 7.8 × 10^8^ CFU/day	CD4 − CD8 − T cells↑, IL-22 and IκBα↑	Yang et al. (114)
*B. licheniformis* and *B. subtilis* mixture	3.9 × 10^8^, 7.8 × 10^8^ or 3.9 × 10^9^ CFU/day	*Lactobacillus gasseri* and *Lactobacillus salivarius* populations↑, goblet cells and MUC2 production↑	Zhang et al. (118)
*B. amyloliquefaciens*	1 × 10^8^ CFU/kg	Intestinal barrier integrity and intestinal antioxidant capacity↑, inflammatory response and enterocyte apoptosis↓	Wang et al. (121)
*B. subtilis*	1 × 10^8^ CFU/kg	IgA-secreting cells and CD3^+^ T cells↑, sIgA↑	Zhang et al. (122)
*B. licheniformis* and *B. subtilis* mixture	3.9 × 10^7^ CFU/mL or 7.8 × 10^7^ CFU/ mL	Foxp3(−)IL-10(+) T cells↑, CD4(+)Foxp3(+) Treg cells↑, CD4(+)IL-10(+) T cells↑	Zhou et al. (123)
*B. subtilis*	2.56 × 10^9^ CFU/kg	*Actinomycetaceae* and *Lachnospiraceae*↓, *Veillonellaceae*, *Bifidobacteriaceae*, Lactobacillaceae↑	Jinno et al. (131)
*B. coagulans*	1 × 10^7^ CFU/g	VH↑, CD↓, IL-10↑, IL-6↓, *Lactobacillus*↑, *E. coli*↓	Liu et al. (142)
*C. butyricum, B. subtilis,* and *B. licheniformis* mixture	5.0 × 10^10^, 5.0 × 10^9^, and 5.0 × 10^9^ CFU/kg, respectively	Ileal apoptotic cells↓, TNF-α and IL-1β↓, butyrate and valerate↑, *Bacillus*↑, intestinal morphology↑	Cao et al. (143)

C3, complement 3; CAT, catalase; CD, crypt depth; GSH-Px, Glutathione peroxidase; GST, glutathione S-transferase; HO-1, heme oxygenase-1; IκBα, NF-kappa-B inhibitor alpha; NQO1, NAD (P)H, quinone oxidoreductase 1; IFN-γ, interferon-γ; IL-1β, Interleukin-1β; IL-6, Interleukin-6; IL-10, Interleukin-10; LZM, lysozyme; MDA, malondialdehyde; MUC2, mucin 2; Nrf2, Nuclear factor erythroid 2-related factor 2; PBD-2, porcine beta defensin-2; RegIIIγ, regenerating islet-derived IIIγ; SGLT1, sodium-glucose cotransporter1; sIgA, secretory immunoglobulin A; SOD, superoxide dismutase; T-AOC, total antioxidant capacity; TNF-α, tumor necrosis factor α; VCR, villus height to crypt depth ratio; VH, villus height; ZO-1, zonula occludens-1; “↑” stands for increase, and “↓” stands for decrease.

Numerous studies have shown that a healthy gut can promote the digestion and absorption of nutrients, thus promoting animal growth (3). Analysis of the existing literatures found that *Bacillus* spp. had good regulatory effects on the intestinal tract of piglets which can promote intestinal development, promote intestinal mucosal barrier, promote intestinal immune function, inhibit inflammatory response, inhibit pathogenic bacteria and regulate intestinal flora homeostasis. Moreover, most of the Bacillus can promote the growth of piglets. Given the close connection between intestinal health and animal growth, we can speculate that *Bacillus* spp. promotes growth by promoting intestinal health of piglets.

## Potential risks and concerns of *Bacillus* spp. as probiotics

Over the past decade, concerns about the overuse of antibiotics have shifted attention to probiotics in the animal feed industry as they can improve growth performance and reduce disease risk (32, 144). The spore-forming *Bacillus* spp. has received extensive scientific and commercial attention, and their beneficial effects have been widely reported and acknowledged (32, 41). However, with a better understanding of their positive role, many questions have been raised about their safety and the relevance of spore formation in the practical application of this class of microorganisms. The first is the safety concerns of the *Bacillus* spp. Some strains of *Bacillus* can cause infections such as bacteremia and endocarditis when they enter the bloodstream (27, 41). For example, A study by Deng et al. (145) who had collected 50 commercial probiotic products and isolated bacillus from the products, which showed that 34 probiotic products (68%) exhibited hemolysis, including 19 human probiotics, 9 animal probiotics, and 6 plant probiotics. 19 of 28 *B. cereus* isolates maintained to exhibit hemolysis after heat treatment. Secondly, pathogenic potential. Some species within the *Bacillus* genus are known to be pathogenic. For example, *Bacillus cereus* is notorious for causing food poisoning, which causes great harm to food safety and animal health by producing enterotoxin and vomitoxin (146, 147). For example, Li et al. (146) showed that piglets received *Bacillus cereus* caused diarrhea, weight loss, and reduced IgG titers of swine fever virus (CSFV) and porcine epidemic diarrhea (PED). The potential for probiotic strains to switch from a beneficial to a pathogenic state is a concern. The third risk factor is antimicrobial and antibiotic resistance issues. There is a worry that the use of *Bacillus* spp. as probiotics could contribute to the spread of antibiotic resistance genes, particularly in the context of their extensive use in the food industry and as a biological control agent in agriculture (41, 148). For example, Deng et al. (145) showed that all 48 *Bacillus* spp. isolates exhibited resistance to lincomycin, and 5 were resistant to tetracycline. Zhu et al. (149) evaluated the safety of 15 strains of *Bacillus cereus* and found that nearly half of the strains carried the antimicrobial resistance gene tet (45). In one strain, tet (45) is located on a mobile genetic element that encodes site-specific recombination mechanisms and is transferred to *Staphylococcus aureus* and *B. subtilis* by electrical transformation. Zhai et al. (150) tested the antimicrobial resistance and antibiotic resistance of 114 isolates of *Bacillus* spp., and the antibiotic susceptibility tests showed that the intrinsic resistance rates of *Bacillus* to ampicillin and penicillin were 80 and 86%, respectively*. Bacillus* strains with acquired antibiotic resistance may spread antibiotic resistance between Bacillus and other clinical pathogens through horizontal gene transfer. Fourth, strain-specificity concerns. As probiotics, the action of *Bacillus* spp. appears to be strain-specific, which means that not all strains will have the same benefits or risks. This adds a layer of complexity to their use and regulation (41). Fifth, lack of standardization. There is a lack of standardization in the production and use of *Bacillus* spp. probiotics, which can lead to variability in efficacy and safety (27). Lastly, regulatory challenges. The regulatory framework for probiotics, including *Bacillus* spp., varies by country and is not always clear, leading to challenges in ensuring the safety and quality of probiotic products (27). In conclusion, *Bacillus* spp. has advantages as well as challenges as an animal probiotic, and safety evaluation should be conducted when using the newly isolated *Bacillus* spp.

## Conclusion

The use of *Bacillus* spp. as probiotics in piglets offers a promising approach to promote growth and enhance intestinal health. The mechanisms by which *Bacillus* spp. exert their beneficial effects include improving growth performance, enhancing intestinal mucosal barrier function, improving intestinal immune function and producing antimicrobial compounds, as well as modulating gut microbiota. Further research is needed to identify the most effective strains and optimal application strategies to maximize the benefits of *Bacillus* spp. as probiotics in piglets.

## Author contributions

XT: Writing – review & editing, Writing – original draft, Visualization, Validation, Supervision, Software, Resources, Project administration, Methodology, Investigation, Funding acquisition, Formal analysis, Data curation, Conceptualization. YZ: Writing – original draft, Formal analysis. KX: Writing – review & editing, Funding acquisition. JZ: Writing – review & editing, Software, Resources.
